# A ‘One Health’ cross-sectional analysis of reports of potential antibiotic resistance cases in international pharmacovigilance databases

**DOI:** 10.3389/fpubh.2026.1799220

**Published:** 2026-05-28

**Authors:** Joseph Mitchell, Pinelopi Lundquist, Camilla Westerberg, Manju Purohit, Cecilia Stålsby Lundborg

**Affiliations:** 1 Health Systems and Policy: Improving Use of Medicines, Department of Global Public Health, Karolinska Institutet, Stockholm, Sweden; 2 Uppsala Monitoring Centre, Uppsala, Sweden; 3 Department of Pathology, Ruxmaniben Deepchand Gardi Medical College, Ujjain, India

**Keywords:** antibiotic resistance, antimicrobial resistance, ecopharmacovigilance, EudraVigilance Veterinary, One Health, pharmacovigilance, VigiBase

## Abstract

**Introduction:**

Antibiotic resistance is a global health problem that requires ‘One Health’ interventions encompassing human, animal, and environmental health. Pharmacovigilance databases have been previously discussed as a potential supplement to traditional antibiotic resistance surveillance, but they have not been explored from a ‘One hHealth’ perspective.

**Methods:**

This study searched VigiBase—the WHO global database of adverse event reports—and EudraVigilance Veterinary, an animal health pharmacovigilance database maintained and managed by the European Medicines Agency, to identify reports related to antibiotic resistance across the ‘One Health’ domains. The searches were performed on all reports received up to February 21st, 2024, for VigiBase and EudraVigilance Veterinary.

**Results:**

A total of 29,667 and 5,217 reports were identified in VigiBase and EudraVigilance Veterinary, respectively. A further search for all antibiotic reports with an environment-related reported adverse event identified 52 reports from both databases, but none of these were related to antibiotic resistance. The reports on potential human and animal antibiotic resistance were increasing in number over time and received from across the globe, despite the differences in scopes of the databases.

**Discussion:**

Pharmacovigilance databases have the capability to capture reports from across the globe. However, limitations remain, particularly a lack of a global database for animal-based pharmacovigilance. For environment-related outcomes there is a scarcity of reports and no database designed to collect them.

## 1 Introduction

Antibiotic resistance is growing global problem that has warranted wide international attention, for instance at the 2024 United Nations Global Assembly (1, 2). The use of antibiotics, a key driver of antibiotic resistance, is increasing in both human and animal health (3, 4). While the impact of antibiotic resistance on human health is well-researched, there has been less focus on animals, where resistance often manifests as a lack of therapeutic effect (5, 6). The environmental impact has received even less attention (5). The presence of antibiotics in the environment is a concern since it can function as reservoirs of antibiotic resistance (7, 8). The quadripartite of the World Health Organization (WHO), World Organization for Animal Health (WOAH), Food and Agriculture Organization (FAO), and United Nations Environment Programme (UNEP) outlined that integrated surveillance systems across ‘one health’ is a priority research agenda in the mitigation of antibiotic resistance (9).

‘One health’ is defined as “an integrated, unifying approach that aims to sustainably balance and optimize the health of the people, animals, and ecosystems. It recognizes that the health of humans, domestic and wild animals, plants, and the wider environment (including ecosystems) are closely linked and interdependent” (10). Due to the inter-related nature of antibiotic resistance, where resistance can transmit between humans, animals, and the environment (5, 11), antibiotic resistance has been labeled as the quintessential ‘one health’ topic (5). There have also been recent calls to incorporate or consider ‘one health’ within pharmacovigilance (12).

Pharmacovigilance, “the science and activities relating to the detection, assessment, understanding and prevention of adverse effects or any other medicine/vaccine related problem” (13), has a potential role across the ‘one health’ domains (12). However, the development of these systems varies across these areas. For human health, there are differing levels of development within the pharmacovigilance system, but there is a well-established global pharmacovigilance network that covers most of the global population (14). However, for animal health, pharmacovigilance systems are less well developed, as action is largely taken by individual countries and regions (15–17). The effects of pharmaceuticals on the environment are not limited to the direct actions of the pharmaceutical ingredients, but there is increasing interest in ecopharmacovigilance, the practice of pharmacovigilance with regards to pharmaceuticals in the environment (18). There are also differences in the targets of the respective surveillance systems, as reporting in human-based pharmacovigilance is based on the effects on humans only, while for animal-based pharmacovigilance it is largely based on the effects on animals and humans. For ecopharmacovigilance the endpoint could be the environment itself or the humans and animals in contact with the environment.

The potential role of pharmacovigilance in combatting antibiotic resistance has been discussed previously (19–25) and descriptive analyses of reports of potential antibiotic resistance cases have been published (21). In particular, the capability of pharmacovigilance to reach under-represented populations has been highlighted as a key role in any utilization (20, 21, 26). However, these works have focused on human health rather than considering a ‘one health’ approach.

## 2 Methods

### 2.1 Data sources

For the human antibiotic cases, VigiBase, the WHO global database of adverse event reports for medicines and vaccines, was used to identify potential reports of antibiotic resistance. VigiBase contains reports from the over 180 members of the WHO Programme of International Drug Monitoring and covers approximately 99% of the global human population (14). VigiBase utilizes the Medical Dictionary for Regulatory Activities (MedDRA®) terminology to code adverse event reports, with analysis typically conducted at the Preferred Term (PT) level.

For animal sources, EudraVigilance Veterinary was used. This database is maintained by the European Medicines Agency, and it contains reports related to veterinary medicinal products authorized for use in the European Economic Area (EEA). The reports come from both inside and outside the EEA, which can occur if the same product is marketed elsewhere outside the EU. All reports, from both within and outside the EEA, were included in the results. Reports in both VigiBase and EudraVigilance Veterinary concern suspected adverse events and reflect only the opinions and observations of the reporters (27). EudraVigilance Veterinary uses terminology from VeDDRA, the Veterinary Dictionary for Drug Regulatory Activities, to report suspected adverse events. VeDDRA is widely used in animal pharmacovigilance databases but has considerably less PTs. The European Medicines Agency also maintains EudraVigilance—a database that contains reports of adverse events related to humans—that forms part of VigiBase and is separate from EudraVigilance Veterinary, with the latter only collecting reports related to animal exposure even if the report subject is a human.

No specific database was identified for environmental pharmacovigilance practices; therefore, VigiBase and EudraVigilance Veterinary were used to identify reports relating to the environment. This was done by identifying PTs within their respective dictionaries related to environmental adverse events.

The Oslo-based WHO Collaborating Centre for Drug Statistics Methodology maintains the Anatomical Therapeutic Chemical (ATC) system for humans and the veterinary counterpart ATCvet. These classification systems were used to identify antibiotics in both databases. They are developed in close association with each other and, with the overlapping nature of medicines used in human and animal-based healthcare, it is possible to link the two systems. This is the recommended methodology for drug utilization studies (28).

### 2.2 Searches

The search of VigiBase was performed on a dataset that included all reports up to April 1st, 2024, and all reports up to February 21st were included. A frozen database was used, as VigiBase is a living database and variations can occur after the initial submission. Automatic deduplication was performed using vigiMatch (29). Reports were identified for inclusion if they included at least one medication from the ATC group J01 (Antibacterials for systemic use) as a suspected or interacting medicine in combination with at least one of a list of previously established MedDRA® PTs (Version 26.1) that are related to antibiotic resistance (Table 1; Supplementary Table S1) (26). Our previous study, which used this group of PTs, separated terms into “Probable” and “Possible” categories, and we identified at least “Likely” cases of antibiotic resistance in 91.4 and 79.3% (95%CI 75.9–82.4) of reports, respectively (26). Both groups of PTs were merged for this study as the “Possible” reports better captured a lack of effect, which is closer in scope to those identified in the animal-based search.

**Table 1:** MedDRA®- and VeDDRA-Preferred Terms used to identify reports of potential antibiotic resistance and environmental exposure in VigiBase and EudraVigilance Veterinary.

Potential Antibiotic Resistance Terms MedDRA
Drug tolerance*, Drug tolerance increased*, Multiple-drug resistance*, Drug resistance*, Pathogen resistance*, Antimicrobial susceptibility test resistant*, Antimicrobial susceptibility test intermediate*, Absence of immediate treatment response, Atypical dose response relationship, Drug effect less than expected, Drug ineffective, Drug ineffective for unapproved indication, Loss of therapeutic response, Missing dose–response relationship, Paradoxical drug reaction, Remission not achieved, Therapeutic product effect decreased, Therapeutic product effect incomplete, Therapeutic product effect variable, Therapeutic product ineffective, Therapeutic product ineffective for unapproved indication, Therapeutic response changed, Therapeutic response decreased, Therapy non-responder, Therapy partial responder, Treatment failure
Potential Antibiotic Resistance Terms VeDDRA
Lack of efficacy
Environmental-related Preferred Terms MedDRA
Environmental exposure, Exposure to chemical pollution, Exposure to contaminated air, Exposure to contaminated water, Exposure to polluted soil, Flooding, Food contamination, Pollution, Poor sanitation, Water pollution, Idiopathic environmental intolerance
Environment-related Preferred Terms VeDDRA
Environmental Incidence

*Indicates Preferred Terms identified as being “Probable” antibiotic resistance cases, the rest were “Possible” in the previous analysis of this search.

To search EudraVigilance Veterinary, the European Medicines Agency performed a search using data up to February 21st, 2024. The search query was for all cases with a product within ATCvet class QJ01 (“Antibacterials for systemic use”) in combination with the PT “Lack of efficacy”. The authors then identified the ATCvet QJ01 codes within each case report. This was done by searching for the trade name; if a specific ATCvet code for that product was not identified, then the ATCvet codes would be taken for each of the active ingredients with an ATCvet code within QJ01. Any report that did not report a medicinal product or active ingredient within ATCvet code QJ01 was excluded. No automatic deduplication was performed on data received from EudraVigilance Veterinary and the report numbers were too large to enable manual deduplication.

To identify potential adverse events related to environmental exposure, a search of MedDRA® and VeDDRA was conducted to identify relevant PTs in each dictionary. A group of environment-related PTs were used to identify potential reports of environmental exposure in VigiBase (Table 1; Supplementary Table S2), and for VedDRA the PT “Environmental incident” was used. These terms were then used to search in VigiBase and EudraVigilance Veterinary, respectively. Initially, reports were screened to include only cases featuring a search term for antibiotic resistance or lack of efficacy, combined with at least one environment-related PT and at least one antibiotic. However, due to a suspected scarcity of results, we also included a search of all reports with an adverse event in combination with an environment-related PT and at least one antibiotic.

The selected search terms for both humans and animals are not synonymous with antibiotic resistance. The broader search terms, which focus more on the lack of effect of antibiotics, may include reports of antibiotic resistance where it has not been coded or when it has not been microbiologically confirmed. However, there are also likely to be cases that reflect other reasons for a lack of effect with antibiotic treatment. These include, but are not limited to, antibiotics not being prescribed incorrectly, adherence issues, product quality issues, and the use of antibiotics against non-susceptible pathogens (19, 26). However, as the potential role of pharmacovigilance databases are to supplement traditional antibiotic resistance surveillance, these reports could indicate resistance that is not captured by traditional surveillance (26). Furthermore, a lack of effect is how most cases of resistance in animals are likely to present (6). We therefore refer to the case reports identified in the search as “potential reports of antibiotic resistance” as, without an individual case-by-case review, it is not possible to accurately state which of these reports does represent a true case of antibiotic resistance, whether it has been microbiologically confirmed or not.

### 2.3 Variables

The country of origin of the reports were extracted and grouped according to their continent, as defined by the UN geoscheme (30). The year of report was calculated according to the first date the report was received in the national pharmacovigilance center for VigiBase reports; if this was not available, the date it was received by VigiBase was used instead. For EudraVigilance Veterinary, the date the report was first received by the database was used.

In VigiBase, each antibiotic in the report was assigned their respective ATC codes. This was done to the fourth-level subgroup (e.g., J01AA – tetracyclines) where possible; if not, the third level of ATC coding was used instead (e.g., J01G – aminoglycoside antibacterials). For EudraVigilance Veterinary reports, each antibiotic in the report was assigned their respective ATCvet codes. Due to the slight discrepancies between the ATC J01 and ATCvet QJ01 classifications, the medicines were also grouped to the third level, which limits the differences between the two systems. As more than one antibiotic can be included in each report, for both VigiBase and EudraVigilance Veterinary, the analysis of antibiotics and ATC codes was done on the number of antibiotics reported rather than the number of reports. The reported antibiotics were also split into two groups: those with a specific antibiotic ingredient reported (e.g., phenoxymethylpenicillin), which were labeled as “antibiotics,” and those reported as groups of antibiotics (i.e., those that matched a third or fourth ATC level term, e.g., fluoroquinolones), which were defined as “antibiotic groups.” Furthermore, the number of reported antibiotics as a compound of ingredients was also calculated for each search.

For the animal reports, the total number of animals affected per each report was used to create a total number of animals affected from all the reports. A count of reports and number of affected animals by species was also calculated.

### 2.4 Data analysis

A descriptive analysis was performed using Microsoft Excel and RStudio. The total number of reports and the respective year and continent of each group of reports were calculated. The percentage for each ATC fourth-level classification was calculated based on the total number of antibiotics reported within each respective group (i.e., humans, animals, and environment). A further cumulative count and percentage was calculated at the third ATC level. For humans and animals, the cumulative counts for each of the third-level groupings were then further divided by continents with both the total number and proportions calculated for each continent for each search. Due to the differences in the scopes of the databases used, no statistical comparison has been made between the databases.

### 2.5 Ethics

Ethical consent was not required for this study as the data in VigiBase and EudraVigilance Veterinary are deidentified before they are available in the database.

## 3 Results

### 3.1 Humans

**Figure 1:**
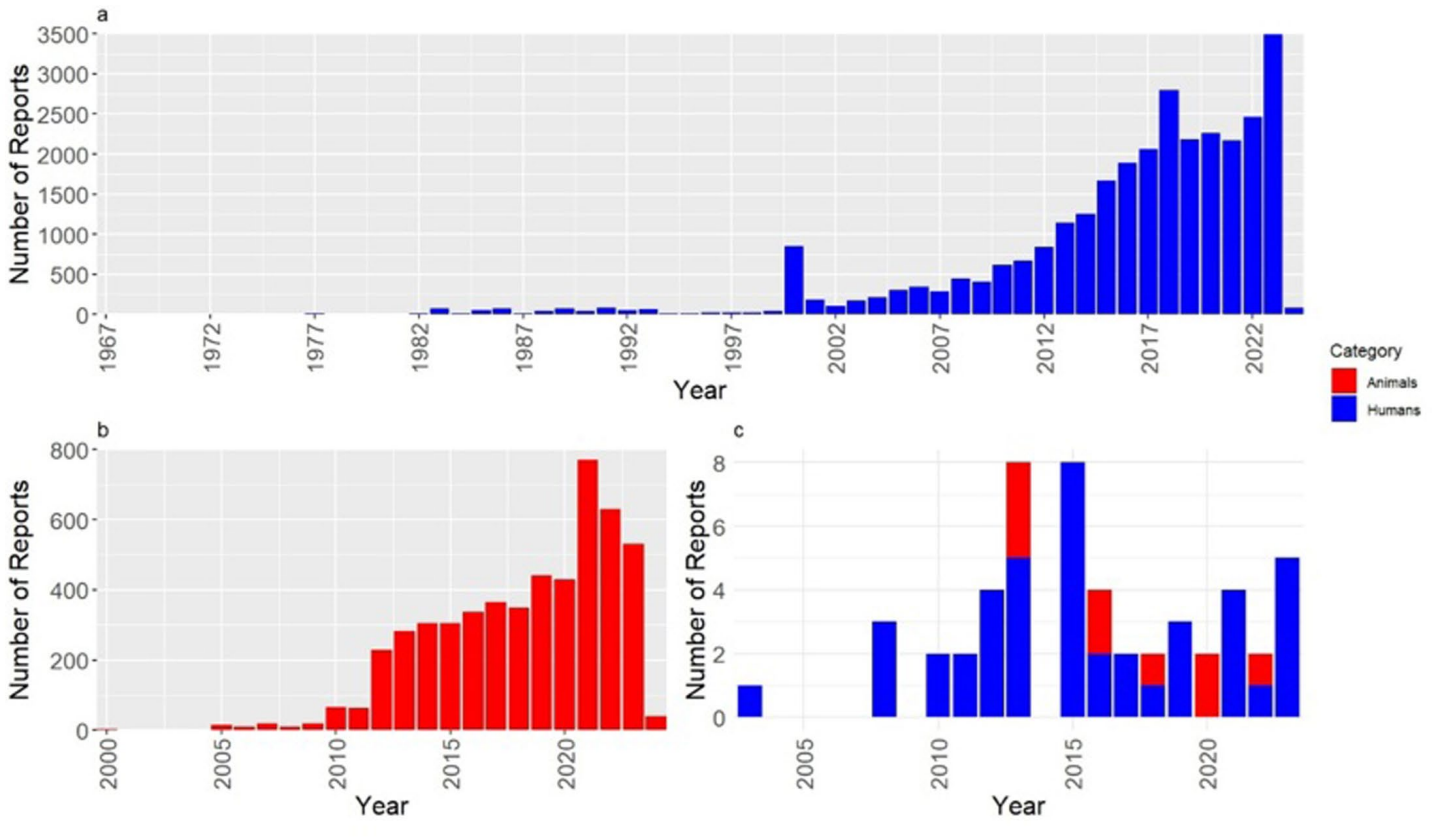
Number of reports, by year, of potential antibiotic resistance received for **(a)** humans in VigiBase, **(b)** animals in EudraVigilance Veterinary and **(c)** environment-related PTs in combination with antibiotics in VigiBase and EudraVigilance Veterinary. The data is up to April 1st, 2024, for VigiBase and February 21st, 2024, for EudraVigilance Veterinary.

A total of 29,667 reports were identified in the VigiBase search for potential reports of human antibiotic resistance. In total, 44,684 antibiotics were reported, with a total of 247 unique antibiotics reported and 15 antibiotic groups. Sixty-one (23.3%) antibiotics (*n* = 60, 24.3%) or antibiotic groups (*n* = 1, 6.7%) were reported as using a combination of ingredients. The number of reports by year is shown in Figure 1 and the first report was received in 1967, with an apparent increase seen from around the year 2000.

Reports were received from 94 countries, with reports from all five UN continents, even if there is a dominance of reporting from the Americas (see Table 2).

**Table 2:** Number and percentage of reports concerning potential antibiotic resistance, categorized by continent (UN Geoscheme) for VigiBase (humans) and EudraVigilance Veterinary (animals).

Continent	Humans				Animals		Environmental	
	Reports	%	Reports	%	Total Animals affected	%	Reports	%
All	29,667		5,217		7,708,327		52	
Africa	841	2.8	52	1.0	336,830	4.4	0	0.0
Americas	18,833	63.5	3,917	75.1	2,894,472	37.5	35	67.3
Asia	2,726	9.2	40	0.8	147,501	1.9	0	0.0
Europe	6,767	22.8	1,158	22.2	4,328,814	56.2	17	32.7
Oceania	500	1.7	46	0.9	666	0.0	0	0.0
Other	0	0.0	4	0.1	44	0.0	0	0.0

For environmental it shows the reports in both VigiBase and EudraVigilance Veterinary that combined for environment-related PTs in combination with antibiotics. The data is up to February 21st, 2024, for VigiBase and EudraVigilance Veterinary. N. B. Percentages are rounded to one decimal point, so percentages may not add up to 100% exactly using the percentages in the table.

### 3.2 Animals

A total of 5,220 reports were received from the EMA. After identifying the ATCvet codes, three were excluded as they did not have an ATC QJ01 code, leaving 5,217 as the final count of included reports. A total of 6,436 antibiotics were reported, with 113 unique antibiotics and two antibiotic groups reported across these reports. Fifty (43.5%) of these antibiotics (*n* = 49, 43.4%) or antibiotic groups (*n* = 1, 50.0%) were reported as a combination of ingredients. The first report was received in 2000, with a general increase in annual reporting approximately 10 years afterwards (see Figure 1).

The reports were regarding 30 different species, with the most common being cattle (*n* = 3,206, 61.5%), dog (*n* = 1,301, 24.9%), and cat (212, 4.1%); however, when looking at the total number of animals affected of these species, they only correspond to 3.2, 0.0, and 0.0% of animals reported, respectively. The most commonly affected animal groups, by total animals affected, are fish (*n* = 6,086,444, 79.5%) and chicken (*n* = 787,871, 10.2%), which account for 0.7 and 1.1% of the total number of reports. The total number of reports and the number of animals affected is shown in Table 3; the full list is in the Supplementary Table S3.

**Table 3:** Total reports and number of animals affected in reports of potential antibiotic resistance in EudraVigilance Veterinary for the species with the ten highest number of reports, up to February 21st, 2024.

Species	Number of reports (*n* = 5,217)	%	Number of animals affected (*n* = 7,708,327)	%
Cattle	3,206	61.5	250,472	3.2
Dog	1,301	24.9	1,387	0.0
Cat	212	4.1	299	0.0
Pig	200	3.8	102,667	1.3
Sheep	67	1.3	3,530	0.0
Chicken	57	1.1	787,871	10.2
Horse	45	0.9	128	0.0
Fish	39	0.7	6,086,444	79.0
Rabbit	29	0.6	41,608	0.5
Turkey	11	0.2	52,074	0.7
Goat	11	0.2	98	0.0
Others	39	0.7	381,749	5.0

N. B. Percentages are rounded to one decimal point, so percentages smaller than this are presented as 0.0%.

Reports were received from 50 countries from all five UN continents, with four reports (0.1%) not having a country of origin listed. Despite the place of surveillance being the EEA, most of the reports were received from countries outside of this region, with 828 (16.3%) from the EEA and 4,365 (83.7%) from outside the EEA. The distribution, by UN Continents, is shown in Table 2; the reports under “Other” are those that did not have a country of origin listed. As with the human data, the highest number of reports originated in the Americas (75.1%); however, when considering the total number of animals involved, Europe reported the highest figure (56.5%).

### 3.3 Environment

Within VigiBase, there were 43 reports identified with an antibiotic and an environment-related PT. But none of these had a PT that was used to identify potential human reports of antibiotic resistance. Within the 43 reports of all environment-related reporting for antibiotics, 60 antibiotics were reported, with 15 unique antibiotics reported. The first report was received in 2003 for VigiBase and 2013 for EudraVigilance Veterinary, with relatively consistent low levels of reporting since then (Figure 1). For EudraVigilance Veterinary, there were nine reports with environmental incidence reported, and none co-reported a lack of effect as a PT. One antibiotic was reported in each report, with six unique antibiotics.

The 43 reports of environment-related terms in combination with antibiotics in VigiBase were received from six countries, with most reports from the Americas (*n* = 35, 81.4%) and the rest from Europe. The nine reports for environmental incidences in combination with antibiotics in EudraVigilance Veterinary were from four countries, with all reports coming from Europe (Table 4). In total there were 26 animals (*n* = 20 pigs, *n* = 3 dog, *n* = 2 cattle, and *n* = 1 chicken) affected in these nine reports. In total, the 52 reports were from eight countries and the distribution across continents shown in Table 2.

**Table 4:** Number of antibiotics reported for each ATC or ATCvet third-level group for human and veterinary potential reports of antibiotics and antibiotics in reports of environment-related PTs in combination with antibiotics reported to VigiBase and EudraVigilance Veterinary, up to February 21st, 2024.

Antibiotics by ATC groups		Human ABR reports		Veterinary ABR reports		Veterinary ABR animals affected		Combined environmental reports for antibiotics	
		*n*	%	*n*	%	*n*	%	*n*	%
Total		44,684		6,436		8,191,609		69	
ATC	J01A	3,579	8.0	1,253	19.5	3,756,621	45.9	5	7.2
	J01B	92	0.2	578	9.0	2,655,610	32.4	0	0.0
	J01C	6,129	13.7	631	9.8	105,776	1.3	6	8.7
	J01D	7,684	17.2	830	12.9	379,018	4.6	4	5.8
	J01E	1,677	3.8	138	2.1	191,655	2.3	4	5.8
	J01F	6,709	15.0	2,410	37.4	441,573	5.4	6	8.7
	J01G	2,487	5.6	98	1.5	22,687	0.3	0	0.0
	J01M	5,469	12.2	249	3.9	308,033	3.8	39	56.5
	J01R	198	0.4	64	1.0	106	0.0	0	0.0
	J01W	72	0.2	0	0.0	0	0.0	0	0.0
	J01X	10,588	23.7	185	2.9	330,530	4.0	5	7.2

### 3.4 Comparison

Of the different antibiotics in the human and veterinary reports, 42 antibiotics and two antibiotic groups were reported for both groups. The distribution of reported antibiotics, and number of animals affected for each antibiotic group, by ATC third level, for each aspect of ‘one health’, is shown in Table 3. The combined environmental reports are both those identified from VigiBase and EudraVigilance Veterinary and are for all environmental reporting with antibiotics. The distribution by the ATC fourth level for each of the searches is shown in the Supplementary Tables S4–S7.

**Figure 2:**
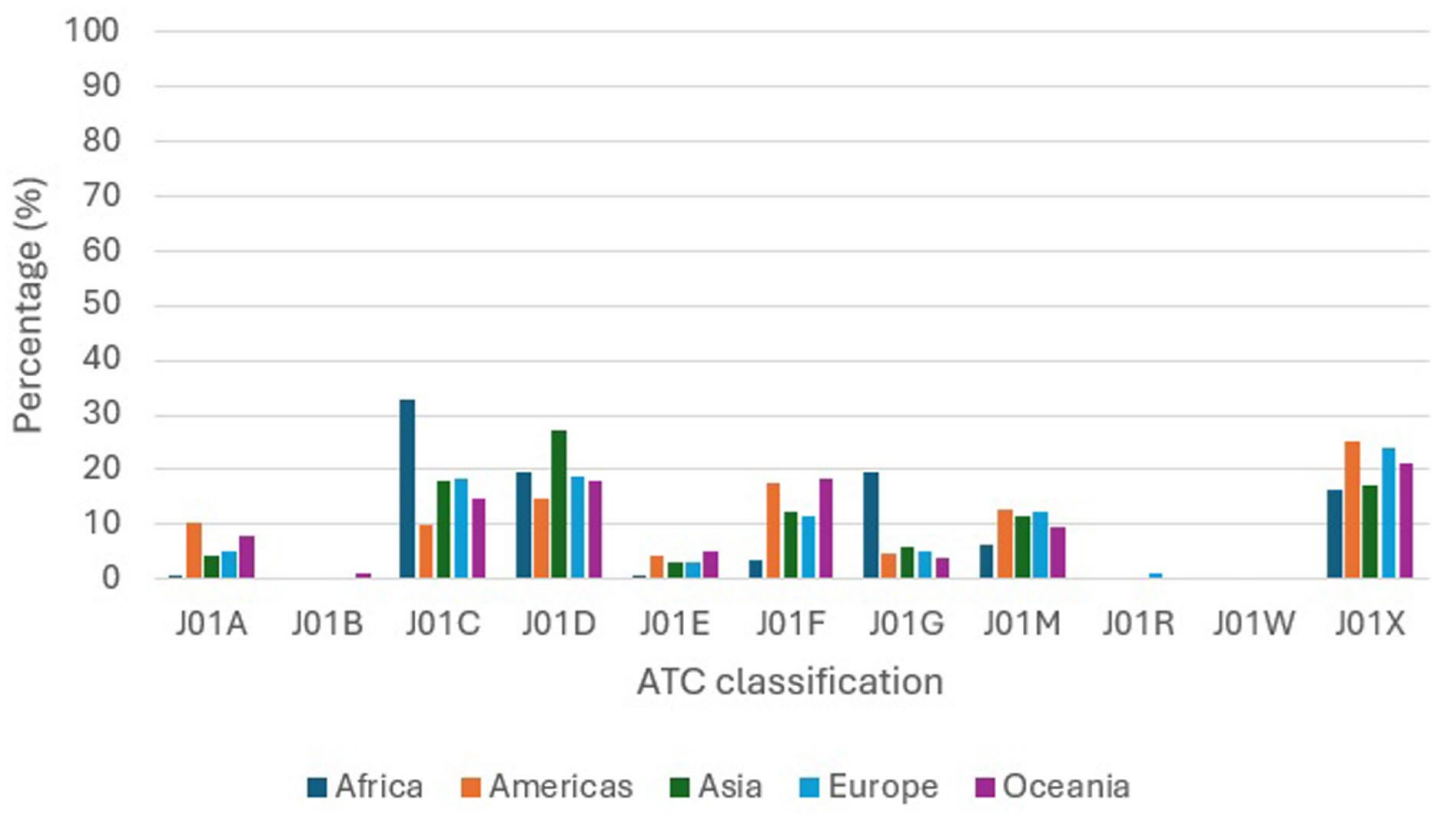
Percentage of antibiotics reported by ATC group third level, for each continent in reports of potential antibiotic resistance in VigiBase, up to April 1st, 2024.

**Figure 3:**
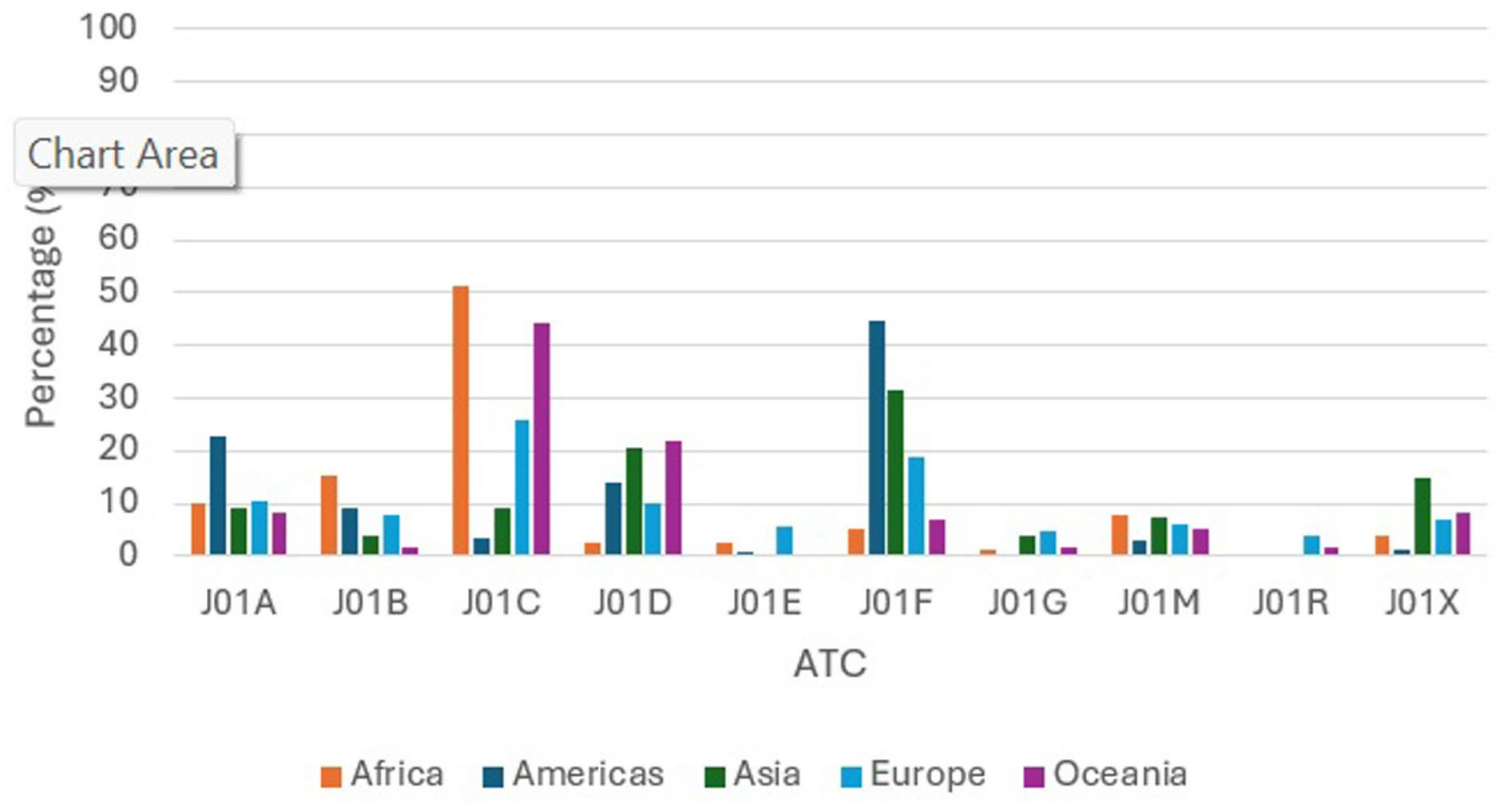
Percentage of reports for each ATCvet group, by third level, for each continent in reports of potential antibiotic resistance in EudraVigilance Veterinary, up to April 1st, 2024.

The distribution of reported antibiotics by ATC third level for each continent is shown in Figure 2 for humans. Generally, the relative distribution of antibiotics reported by continent is quite similar, i.e., the ATC classes with higher percentages of reporting in one continent are likely to be high in the other continents too. This is particularly true for continents where the total number of reports is high. Figure 3 shows the distribution of reported antibiotics by ATC third level grouped by the continent for animals. Compared to humans, there appears to be greater variance between continents for the relative number of antibiotics reported by ATC classification at the third level. However, it is important to consider that the total number of reports is smaller, and very small for some of the continents. This increasing variation is exacerbated further when considering the total number of animals affected (Supplementary Figure S1).

## 4 Discussion

This study analyzed potential reports of antibiotic resistance in pharmacovigilance databases from a ‘one health’ perspective. The reporting of potential antibiotic resistance reports in EudraVigilance Veterinary and VigiBase, for animals and humans, respectively, is increasing. There is, however, a scarcity of environment-based reporting.

The searches used to identify potential reports of antibiotic resistance showed that the total number of reports received each year is generally increasing for humans and animals. We have not shown these increases are relative to the whole databases or to the total antibiotic-related reports, as this data was not available to us for both databases. However, the number of reports submitted to pharmacovigilance databases is generally increasing (31, 32). The role of pharmacovigilance as a tool to help combat antibiotic resistance is still in the early stages of exploration (20, 21, 26) and, similarly, pharmacovigilance from a ‘one health’ perspective is a relatively new concept (12). To the best of the authors’ knowledge, this study is the first to combine the two concepts. To address the threat faced by antibiotic resistance there is a need to adopt a ‘one health’ approach (5, 9, 11). This should be recommended if incorporating pharmacovigilance as a supplement to traditional antibiotic resistance surveillance. How pharmacovigilance can best complement traditional antibiotic resistance surveillance is not yet known. Potential uses include monitoring for a local or global increase in reports of potential antibiotic resistance that triggers further microbiological investigation or it may even be used alongside data collection systems for antibiotic consumption and resistance levels to improve real-time surveillance (19). For humans, international pharmacovigilance databases have been shown to accumulate reports that could otherwise be underrepresented in traditional antibiotic resistance, with reports from low-resource settings and non-healthcare professionals (26). While, in animal health, the reports are captured as lack of effect, which matches how cases are typically identified in clinical practice (6). The imbalance of research focusing on human, animal, and environmental health for antibiotic resistance has previously been discussed (5) and this study mirrors this disparity. Despite the differences in the scope of the global VigiBase and regional EudraVigilance Veterinary, they have both captured reports globally. The imbalance of the scopes of each database negates the validity of over-interpreting any comparisons, such as the distribution of antibiotics reported. Furthermore, VigiBase benefits from an automatic deduplication tool, whereas EudraVigilance Veterinary does not. This further hinders any comparison of over-time trends between the databases. However, it is notable that reports were received across the ATC classifications in both human and animal health. There was also reasonable consistency, within human-based data, in particular, of the relative geographical spread of antibiotics reported when classified by the ATC third level.

The total number of reports in VigiBase is much higher than in EudraVigilance Veterinary; however, when considering the affected population, the number in EudraVigilance Veterinary dwarfs that of VigiBase. There was also a great variability in how reporting differed between species, with fish accounting for only 0.7% of reports but 79.0% of individual animals affected. This highlights the reporting of different treatment types, i.e., via individually dosed medicines akin to human healthcare and administration via metaphylactic means, such as in feed or water (6). This disparity is due to the wide variety of species and to the different reasons for keeping and caring for animals, with both treatment of individual animals (such as companion animals) and large groups of animals (such as food-producing animals) (33). These findings may not be unexpected but still highlight the importance of considering the types of animal species used in future comparisons and how this can affect our interpretation of them.

Unsurprisingly, the number of different antibiotics and antibiotic groups in reports from EudraVigilance Veterinary were less than that of reports in VigiBase, at 115 and 262, respectively. There was a considerable overlap of antibiotics reported, with 44 (42 antibiotics and two antibiotic groups) reported in both VigiBase and EudraVigilance Veterinary. It was also noted that the percentage of different antibiotics reported as combinations was higher in the veterinary database than the human equivalent, at 43.5 and 23.3%, respectively. Reporting into EudraVigilance Veterinary is limited to products that are authorized for veterinarian use in the European Union (EU) (34). This does not cover all medicines used in animal healthcare. The established prescribing cascade guidelines help veterinarians to prescribe suitable products that are not authorized for veterinary use (33, 34). This applies to antibiotics too, and there are special considerations and further categorization of antibiotics to promote responsible use (33). The EU has quite stringent practices when it comes to antibiotic use in animal healthcare (33), and it is likely that the differences between the total number of reports and the number of animals affected by continent reflects the scope of the EudraVigilance Veterinary database and reporting practices rather than different prescribing practices. A more comprehensive surveillance of products used in veterinary healthcare may benefit from a global pharmacovigilance network and database akin to the WHO Programme of International Drug Monitoring and VigiBase. This would likely increase global capabilities to perform animal-based pharmacovigilance activities, as well as facilitating the use of pharmacovigilance across the ‘one health’ concept.

The scarcity of environmental-related reporting is perhaps expected, despite the surge in interest in the effects of antibiotics on the environment. The endpoint of the databases searched here are of humans and animals and not the environment itself. Therefore, there is an extra step of suspicion required in suspecting the adverse event is not only related to an exposure to medicine but also that it was related to an environmental exposure, which in most cases would be a rare occurrence (18). Typically, the levels and any potential effects of environmental antibiotic accumulation are gathered in individual literature reports (18). Even though the equivalent endpoint of an adverse event in the environment is less clear, it may be an important starting point to accumulate the information in one place. Examples could simply be evidence of the accumulation of antibiotics in the environment or, perhaps more realistically, of occurrences of concentrations above the Predicted No-Effect Concentration (PNEC) (35, 36).

The reports of VigiBase and EudraVigilance Veterinary both represent suspected adverse events only and that the likelihood of an adverse event being related to the medicinal product is not equal between reports. This is an important limitation to consider, especially as there are multiple reasons for a lack of effect in antibiotics, of which antibiotic resistance is one. However, this search has recently been analyzed on a case-by-case basis in VigiBase (26), and lack of effect is how antibiotic resistance would be discovered in veterinary care in most cases (6), even if it is poorly reported (34). To enhance the future utility of pharmacovigilance databases, it may be beneficial to expand medical dictionaries—particularly VeDDRA—to more clearly define potential causes of lack of efficacy.

## 5 Conclusion

This study is the first to consider the role of pharmacovigilance in antibiotic resistance surveillance from a ‘One Health’ perspective. Pharmacovigilance databases from human and animal health have the capacity to capture reports of potential antibiotic resistance from across the globe. The number of reports accumulated annually is generally increasing. However, limitations remain as to the scope of the databases within animal healthcare, as there is no global coordination and environment-based reporting across database is still scarce.

## Data Availability

The datasets presented in this article are not readily available because the data that support the findings of this study are not publicly available. Access to the VigiBase data is restricted based on the conditions for access within the WHO Programme for International Drug Monitoring. Subject to these conditions, some of the data is available from the authors on reasonable request. Data from EudraVigilance Veterinary was requested and received from the European Medicines Agency. Requests to access the datasets should be directed to joseph.mitchell@ki.se.
