# Supplementary material for: A ‘One Health’ cross-sectional analysis of reports of potential antibiotic resistance cases in international pharmacovigilance databases
Source: Front Public Health. 2026 May 28;14:1799220. doi: 10.3389/fpubh.2026.1799220 (PMC13253955; doi:10.3389/fpubh.2026.1799220)
Supplement: Supplementary file 1 [file Supplementary_file_1.docx]

Supplementary Material

# Supplementary Tables

Supplementary Table 1. Search Query Used to Identify Potential Reports of Antibiotic Resistance in VigiBase

| MedDRA Version | 26.1 |
| --- | --- |
| Dataset date | 2024-04-01 |
| Medicine | Any drug in J01A,B,C,D,E,F,G,M,R,W, or X |
| Basis of Suspicion of the Medicine | Suspected/interacting |
| Reaction | Drug tolerance, Drug tolerance increased, Multiple-drug resistance, Drug resistance, Pathogen resistance, Antimicrobial susceptibility test resistant, Antimicrobial susceptibility test intermediate, Absence of immediate treatment response, Atypical dose response relationship, Drug effect less than expected, Drug ineffective, Drug ineffective for unapproved indication, Loss of therapeutic response, Missing dose response relationship, Paradoxical drug reaction, Remission not achieved, Therapeutic product effect decreased, Therapeutic product effect incomplete, Therapeutic product effect variable, Therapeutic product ineffective, Therapeutic product ineffective for unapproved indication, Therapeutic response changed, Therapeutic response decreased, Therapy non-responder, Therapy partial responder, Treatment failure |
| Period | Up to 2024-02-21 |
| Region | All |
| Other | Frozen dataset, de-duplicated |

Supplementary Table 2. Search Query Used to Identify Environment-Related Reports With and Without Terms Related to Antibiotic Resistance in VigiBase

| MedDRA Version | 26.1 |
| --- | --- |
| Dataset date | 2024-04-01 |
| Medicine | Any drug in J01A,B,C,D,E,F,G,M,R,W, or X |
| Basis of Suspicion of the Medicine | Suspected/interacting |
| Reaction | Environmental exposure, Exposure to chemical pollution, Exposure to contaminated air, Exposure to contaminated water, Exposure to polluted soil, Flooding, Food contamination, Pollution, Poor sanitation, Water pollution, Idiopathic environmental intolerance |
| Period | Up to 2024-02-21 |
| Region | All |
| Other | Frozen dataset, de-duplicated |

Supplementary Table 3. Number of reports and total number of animals affected in reports of potential antibiotic resistance in EudraVigilance Veterinary by species, up to February 21st, 2024.

| **Species** | **Number of Reports**  **(n=5,217)** | **%** | **Number of animals affected**  **(n=7,708,327)** | **%** |
| --- | --- | --- | --- | --- |
| Cattle | 3,206 | 61.5 | 250,472 | 3.2 |
| Dog | 1,301 | 24.9 | 1,387 | 0.0 |
| Cat | 212 | 4.1 | 299 | 0.0 |
| Pig | 200 | 3.8 | 102,667 | 1.3 |
| Sheep | 67 | 1.3 | 3,530 | 0.0 |
| Chicken | 57 | 1.1 | 787,871 | 10.2 |
| Horse | 45 | 0.9 | 128 | 0.0 |
| Fish | 39 | 0.7 | 6,086,444 | 79.0 |
| Rabbit | 29 | 0.6 | 41,608 | 0.5 |
| Turkey | 11 | 0.2 | 52,074 | 0.7 |
| Goat | 11 | 0.2 | 98 | 0.0 |
| Duck | 9 | 0.2 | 37,820 | 0.5 |
| Pheasant | 4 | 0.1 | 1,440 | 0.0 |
| Other | 4 | 0.1 | 6 | 0.0 |
| Other birds | 3 | 0.1 | 332,260 | 4.3 |
| Alpaca | 3 | 0.1 | 3 | 0.0 |
| Quail | 2 | 0.0 | 9,999 | 0.1 |
| Donkey | 2 | 0.0 | 2 | 0.0 |
| Guinea fowl | 1 | 0.0 | 100 | 0.0 |
| Other cervids | 1 | 0.0 | 60 | 0.0 |
| Partridge | 1 | 0.0 | 30 | 0.0 |
| Guinea pig | 1 | 0.0 | 7 | 0.0 |
| Buffalo | 1 | 0.0 | 6 | 0.0 |
| Other bovine | 1 | 0.0 | 6 | 0.0 |
| Other rodents | 1 | 0.0 | 4 | 0.0 |
| Pigeon | 1 | 0.0 | 2 | 0.0 |
| Ferret | 1 | 0.0 | 1 | 0.0 |
| Hedgehog | 1 | 0.0 | 1 | 0.0 |
| Monkey | 1 | 0.0 | 1 | 0.0 |
| Snake | 1 | 0.0 | 1 | 0.0 |

## Humans

Supplementary Table 4. Counts and Percentages of ATC groups of antibiotics reported in potential cases of antibiotic resistance in humans in VigiBase, up to February 21^st^, 2024.

| **ATC Classification** |  |  | Count | Percentage |
| --- | --- | --- | --- | --- |
| **Total** |  |  | **44,684** |  |
| **J01 – Antibacterials for systemic use** |  |  | 0 | 0.0 |
|  | **J01A - Tetracyclines** |  | 0 | 0.0 |
|  |  | J01AA – Tetracyclines | 3,579 | 8.0 |
|  | **J01B - Amphenicols** |  | 0 | 0.0 |
|  |  | J01BA – Amphenicols | 92 | 0.2 |
|  | **J01C - Beta-Lactam Antibacterials, Penicillins** |  | 101 | 0.2 |
|  |  | J01CA - Penicillins with extended spectrum | 2,277 | 5.1 |
|  |  | J01CE - Beta-lactamase sensitive penicillins | 369 | 0.8 |
|  |  | J01CF - Beta-lactamase resistant penicillins | 315 | 0.7 |
|  |  | J01CG - Beta-lactamase inhibitors | 63 | 0.1 |
|  |  | J01CR - Combinations of penicillins, incl. beta-lactamase inhibitors | 3,004 | 6.7 |
|  | **J01D – Other Beta-Lactam Antibacterials** |  | 3 | 0 |
|  |  | J01DB - First-generation cephalosporins | 969 | 2.2 |
|  |  | J01DC - Second-generation cephalosporins | 777 | 1.7 |
|  |  | J01DD - Fourth-generation cephalosporins | 2,717 | 6.1 |
|  |  | J01DE - Fourth-generation cephalosporins | 539 | 1.2 |
|  |  | J01DF - Monobactams | 192 | 0.4 |
|  |  | J01DH - Carbapenems | 2,177 | 4.9 |
|  |  | J01DI - Other cephalosporins and penems | 310 | 0.7 |
|  | **J01E – Sulfonamides And Trimethoprim** |  | 2 | 0.0 |
|  |  | J01EA - Trimethoprim and derivatives | 105 | 0.2 |
|  |  | J01EB - Short-acting sulfonamides | 30 | 0.1 |
|  |  | J01EC - Intermediate-acting sulfonamides | 80 | 0.2 |
|  |  | J01ED - Long-acting sulfonamides | 4 | 0.0 |
|  |  | J01EE - Combinations of sulfonamides and trimethoprim, incl. derivatives | 1,456 | 3.3 |
|  | **J01F – Macrolides, Lincosamides and Streptogramins** |  | 0 | 0.0 |
|  |  | J01FA - Macrolides | 5,044 | 11.3 |
|  |  | J01FF - Lincosamides | 1,614 | 3.6 |
|  |  | J01FG - Streptogramins | 51 | 0.1 |
|  | **J01G – Aminoglycoside Antibacterials** |  | 1 | 0.0 |
|  |  | J01GA -Streptomycins | 132 | 0.3 |
|  |  | J01GB - Other aminoglycosides | 2,354 | 5.3 |
|  | **J01M – Quinoline Antibacterials** |  | 5 | 0.0 |
|  |  | J01MA - Fluoroquinolones | 5,447 | 12.2 |
|  |  | J01MB - Other quinolones | 17 | 0.0 |
|  | **J01R – Combinations Of Antibacterials** |  | 0 | 0.0 |
|  |  | J01RA - Combinations of antibacterials | 198 | 0.4 |
|  | **J01W- Herbal Antibacterials and Antiinfectives For Systemic Use** |  | 15 | 0.0 |
|  |  | J01WA - Herbal antibacterials for systemic use | 15 | 0.0 |
|  |  | J01WB - Herbal urinary antiseptics and anti-infectives | 42 | 0.1 |
|  | **J01X – Other Antibacterials** |  | 0 | 0.0 |
|  |  | J01XA - Glycopeptide antibacterials | 3,479 | 7.8 |
|  |  | J01XB - Polymyxins | 496 | 1.1 |
|  |  | J01XC - Steroid antibacterials | 83 | 0.2 |
|  |  | J01XD - Imidazole derivatives | 2,536 | 5.7 |
|  |  | J01XE - Nitrofuran derivatives | 326 | 0.7 |
|  |  | J01XX - Other antibacterials | 3,668 | 8.2 |

## Animals

Supplementary Table 5. Counts and Percentages of ATCvet groups of antibiotics reported in potential cases of antibiotic resistance in animals in EudraVigilance Veterinary, up to February 21st, 2024.

| **ATCvet Classification** | | | **Count by report (n=6,436)** | **Percentage by report** | **Count by affected animals (n=8,191,610** | **Percentage of cases** |
| --- | --- | --- | --- | --- | --- | --- |
| **QJ01 – Antibacterials for systemic use** | **QJ01A - Tetracyclines** |  | 0 | 0.0 | 0 | 0.0 |
|  |  | QJ01AA – Tetracyclines | 1,253 | 19.5 | 3,756,621 | 45.9 |
|  | **QJ01B - Amphenicols** |  | 0 | 0.0 | 0 | 0.0 |
|  |  | QJ01BA – Amphenicols | 578 | 9.0 | 2,655,610 | 32.4 |
|  | **QJ01C - Beta-Lactam Antibacterials, Penicillins** |  | 5 | 0.1 | 80 | 0.0 |
|  |  | QJ01CA - Penicillins with extended spectrum | 173 | 2.7 | 72,690 | 0.9 |
|  |  | QJ01CE - Beta-lactamase sensitive penicillins | 206 | 3.2 | 30,706 | 0.4 |
|  |  | QJ01CF - Beta-lactamase resistant penicillins | 19 | 0.3 | 183 | 0.0 |
|  |  | QJ01CG - Beta-lactamase inhibitors | 0 | 0.0 | 0 | 0.0 |
|  |  | QJ01CR - Combinations of penicillins, incl. beta-lactamase inhibitors | 228 | 3.5 | 2,117 | 0.0 |
|  | **QJ01D – Other Beta-Lactam Antibacterials** |  | 0 | 0.0 | 0 | 0.0 |
|  |  | QJ01DB - First-generation cephalosporins | 99 | 1.5 | 194 | 0.0 |
|  |  | QJ01DC - Second-generation cephalosporins | 0 | 0.0 | 0 | 0.0 |
|  |  | QJ01DD - Fourth-generation cephalosporins | 686 | 10.7 | 378,502 | 4.6 |
|  |  | QJ01DE - Fourth-generation cephalosporins | 45 | 0.7 | 322 | 0.0 |
|  |  | QJ01DF - Monobactams | 0 | 0.0 | 0 | 0.0 |
|  |  | QJ01DH - Carbapenems | 0 | 0.0 | 0 | 0.0 |
|  |  | QJ01DI - Other cephalosporins and penems | 0 | 0.0 | 0 | 0.0 |
|  | **QJ01E – Sulfonamides And Trimethoprim** |  | 0 | 0.0 | 0 | 0.0 |
|  |  | QJ01EA - Trimethoprim and derivatives | 0 | 0.0 | 0 | 0.0 |
|  |  | QJ01EQ – Sulfonamides | 25 | 0.4 | 6,708 | 0.1 |
|  |  | QJ01EW – Combinations of sulfonamides and trimethoprim, incl. derivatives | 113 | 1.8 | 184,947 | 2.3 |
|  | **QJ01F – Macrolides, Lincosamides and Streptogramins** |  | 0 | 0.0 | 0 | 0.0 |
|  |  | QJ01FA - Macrolides | 2,350 | 36.5 | 341,030 | 4.2 |
|  |  | QJ01FF - Lincosamides | 60 | 0.9 | 100,543 | 1.2 |
|  |  | QJ01FG - Streptogramins | 0 | 0.0 | 0 | 0.0 |
|  | **QJ01G – Aminoglycoside Antibacterials** |  | 0 | 0.0 | 0 | 0.0 |
|  |  | QJ01GA -Streptomycins | 8 | 0.1 | 735 | 0.0 |
|  |  | QJ01GB - Other aminoglycosides | 90 | 1.4 | 21,952 | 0.3 |
|  | **QJ01M – Quinoline Antibacterials** |  | 0 | 0.0 | 0 | 0.0 |
|  |  | QJ01MA - Fluoroquinolones | 235 | 3.7 | 159,907 | 2.0 |
|  |  | QJ01MB - Other quinolones | 14 | 0.2 | 148,126 | 1.8 |
|  |  | QJ01MQ - Quinoxalines |  |  |  |  |
|  | **QJ01R – Combinations Of Antibacterials** |  | 0 | 0.0 | 0 | 0.0 |
|  |  | QJ01RA - Combinations of antibacterials | 60 | 0.9 | 98 | 0.0 |
|  |  | QJ01RV – Combinations of antibacterials and other substances | 4 | 0.1 | 8 | 0.0 |
|  | **QJ01X – Other Antibacterials** |  | 0 | 0.0 | 0 | 0.0 |
|  |  | QJ01XA - Glycopeptide antibacterials | 0 | 0.0 | 0 | 0.0 |
|  |  | QJ01XB - Polymyxins | 25 | 0.4 | 170,804 | 2.1 |
|  |  | QJ01XC - Steroid antibacterials | 1 | 0.0 | 1 | 0.0 |
|  |  | QJ01XD - Imidazole derivatives | 89 | 1.4 | 94 | 0.0 |
|  |  | QJ01XE - Nitrofuran derivatives | 0 | 0.0 | 0 | 0.0 |
|  |  | QJ01XG - Pleuromutilins | 35 | 0.5 | 66,658 | 0.8 |
|  |  | QJ01XX - Other antibacterials | 35 | 0.5 | 92,973 | 1.1 |

## Environment

Supplementary Table 6. Counts and Percentages of ATC groups of antibiotics reported in environment-related reports in combination with antibiotics in VigiBase, up to February 21^st^, 2024.

| **ATC Classification** |  |  | Count | Percentage |
| --- | --- | --- | --- | --- |
| **Total** |  |  | **60** |  |
|  | **J01A - Tetracyclines** |  | 0 | 0.0 |
|  |  | J01AA – Tetracyclines | 2 | 3.3 |
|  | **J01B - Amphenicols** |  | 0 | 0.0 |
|  |  | J01BA – Amphenicols | 0 | 0.0 |
|  | **J01C - Beta-Lactam Antibacterials, Penicillins** |  | 0 | 0.0 |
|  |  | J01CA - Penicillins with extended spectrum | 3 | 5.0 |
|  |  | J01CE - Beta-lactamase sensitive penicillins | 1 | 1.7 |
|  |  | J01CF - Beta-lactamase resistant penicillins | 0 | 0.0 |
|  |  | J01CG - Beta-lactamase inhibitors | 0 | 0.0 |
|  |  | J01CR - Combinations of penicillins, incl. beta-lactamase inhibitors | 0 | 0.0 |
|  | **J01D – Other Beta-Lactam Antibacterials** |  | 0 | 0.0 |
|  |  | J01DB - First-generation cephalosporins | 0 | 0.0 |
|  |  | J01DC - Second-generation cephalosporins | 3 | 5.0 |
|  |  | J01DD - Fourth-generation cephalosporins | 0 | 0.0 |
|  |  | J01DE - Fourth-generation cephalosporins | 0 | 0.0 |
|  |  | J01DF - Monobactams | 0 | 0.0 |
|  |  | J01DH - Carbapenems | 0 | 0.0 |
|  |  | J01DI - Other cephalosporins and penems | 0 | 0.0 |
|  | **J01E – Sulfonamides And Trimethoprim** |  | 0 | 0.0 |
|  |  | J01EA - Trimethoprim and derivatives | 0 | 0.0 |
|  |  | J01EB - Short-acting sulfonamides | 0 | 0.0 |
|  |  | J01EC - Intermediate-acting sulfonamides | 0 | 0.0 |
|  |  | J01ED - Long-acting sulfonamides | 0 | 0.0 |
|  |  | J01EE - Combinations of sulfonamides and trimethoprim, incl. derivatives | 4 | 6.7 |
|  | **J01F – Macrolides, Lincosamides and Streptogramins** |  | 0 | 0.0 |
|  |  | J01FA - Macrolides | 3 | 5.0 |
|  |  | J01FF - Lincosamides | 0 | 0.0 |
|  |  | J01FG - Streptogramins | 0 | 0.0 |
|  | **J01G – Aminoglycoside Antibacterials** |  | 0 | 0.0 |
|  |  | J01GA -Streptomycins | 0 | 0.0 |
|  |  | J01GB - Other aminoglycosides | 0 | 0.0 |
|  | **J01M – Quinoline Antibacterials** |  | 0 | 0.0 |
|  |  | J01MA - Fluoroquinolones | 39 | 65.0 |
|  |  | J01MB - Other quinolones | 0 | 0.0 |
|  | **J01R – Combinations Of Antibacterials** |  | 0 | 0.0 |
|  |  | J01RA - Combinations of antibacterials | 0 | 0.0 |
|  | **J01W- Herbal Antibacterials and Antiinfectives For Systemic Use** |  | 0 | 0.0 |
|  |  | J01WA - Herbal antibacterials for systemic use | 0 | 0.0 |
|  |  | J01WB - Herbal urinary antiseptics and anti-infectives | 0 | 0.0 |
|  | **J01X – Other Antibacterials** |  | 0 | 0.0 |
|  |  | J01XA - Glycopeptide antibacterials | 1 | 1.7 |
|  |  | J01XB - Polymyxins | 1 | 1.7 |
|  |  | J01XC - Steroid antibacterials | 0 | 0.0 |
|  |  | J01XD - Imidazole derivatives | 3 | 5.0 |
|  |  | J01XE - Nitrofuran derivatives | 0 | 0.0 |
|  |  | J01XX - Other antibacterials | 0 | 0.0 |

Supplementary Table 7. Counts and Percentages of ATC groups of antibiotics reported in environment-related reports in combination with antibiotics in EudraVigilance Veterinary, up to February 21^st^, 2024.

| **ATC Classification** | | | **Count by report** | **Percentage by report** | **Count by affected animals** | **Percentage of cases** |
| --- | --- | --- | --- | --- | --- | --- |
|  |  | | **9** |  | **26** |  |
| **QJ01 – Antibacterials for systemic use** |  | | **QJ01 – Antibacterials for systemic use** |  |  |  |
|  | **QJ01A - Tetracyclines** |  | 0 | 0.0 | 0 | 0.0 |
|  |  | J01AA – Tetracyclines | 3 | 33.3 | 3 | 11.5 |
|  | **QJ01B - Amphenicols** |  | 0 | 0.0 | 0 | 0.0 |
|  |  | J01BA – Amphenicols | 0 | 0.0 | 0 | 0.0 |
|  | **QJ01C - Beta-Lactam Antibacterials, Penicillins** |  | 0 | 0.0 | 0 | 0.0 |
|  |  | J01CA - Penicillins with extended spectrum | 0 | 0.0 | 0 | 0.0 |
|  |  | J01CE - Beta-lactamase sensitive penicillins | 2 | 22.2 | 2 | 7.7 |
|  |  | J01CF - Beta-lactamase resistant penicillins | 0 | 0.0 | 0 | 0.0 |
|  |  | J01CG - Beta-lactamase inhibitors | 0 | 0.0 | 0 | 0.0 |
|  |  | J01CR - Combinations of penicillins, incl. beta-lactamase inhibitors | 0 | 0.0 | 0 | 0.0 |
|  | **QJ01D – Other Beta-Lactam Antibacterials** |  | 0 | 0.0 | 0 | 0.0 |
|  |  | J01DB - First-generation cephalosporins | 0 | 0.0 | 0 | 0.0 |
|  |  | J01DC - Second-generation cephalosporins | 0 | 0.0 | 0 | 0.0 |
|  |  | J01DD - Fourth-generation cephalosporins | 1 | 11.1 | 0 | 0.0 |
|  |  | J01DE - Fourth-generation cephalosporins | 0 | 0.0 | 0 | 0.0 |
|  |  | J01DF - Monobactams | 0 | 0.0 | 0 | 0.0 |
|  |  | J01DH - Carbapenems | 0 | 0.0 | 0 | 0.0 |
|  |  | J01DI - Other cephalosporins and penems | 0 | 0.0 | 0 | 0.0 |
|  | **QJ01E – Sulfonamides And Trimethoprim** |  | 0 | 0.0 | 0 | 0.0 |
|  |  | J01EA - Trimethoprim and derivatives | 0 | 0.0 | 0 | 0.0 |
|  |  | J01EQ – Sulfonamides | 0 | 0.0 | 0 | 0.0 |
|  |  | J01EW – Combinations of sulfonamides and trimethoprim, incl. derivatives | 0 | 0.0 | 0 | 0.0 |
|  | **QJ01F – Macrolides, Lincosamides and Streptogramins** |  | 0 | 0.0 | 0 | 0.0 |
|  |  | J01FA - Macrolides | 3 | 33.3 | 21 | 80.8 |
|  |  | J01FF - Lincosamides | 0 | 0.0 | 0 | 0.0 |
|  |  | J01FG - Streptogramins | 0 | 0.0 | 0 | 0.0 |
|  | **QJ01G – Aminoglycoside Antibacterials** |  | 0 | 0.0 | 0 | 0.0 |
|  |  | J01GA -Streptomycins | 0 | 0.0 | 0 | 0.0 |
|  |  | J01GB - Other aminoglycosides | 0 | 0.0 | 0 | 0.0 |
|  | **QJ01M – Quinoline Antibacterials** |  | 0 | 0.0 | 0 | 0.0 |
|  |  | J01MA - Fluoroquinolones | 0 | 0.0 | 0 | 0.0 |
|  |  | J01MB - Other quinolones | 0 | 0.0 | 0 | 0.0 |
|  |  | J01MQ - Quinoxolines |  |  |  |  |
|  | **QJ01R – Combinations Of Antibacterials** |  | 0 | 0.0 | 0 | 0.0 |
|  |  | J01RA - Combinations of antibacterials | 0 | 0.0 | 0 | 0.0 |
|  |  | J01RV – Combinations of antibacterials and other substances | 0 | 0.0 | 0 | 0.0 |
|  | **QJ01X – Other Antibacterials** |  | 0 | 0.0 | 0 | 0.0 |
|  |  | J01XA - Glycopeptide antibacterials | 0 | 0.0 | 0 | 0.0 |
|  |  | J01XB - Polymyxins | 0 | 0.0 | 0 | 0.0 |
|  |  | J01XC - Steroid antibacterials | 0 | 0.0 | 0 | 0.0 |
|  |  | J01XD - Imidazole derivatives | 0 | 0.0 | 0 | 0.0 |
|  |  | J01XE - Nitrofuran derivatives | 0 | 0.0 | 0 | 0.0 |
|  |  | J01XG - Pleuromutilins | 0 | 0.0 | 0 | 0.0 |
|  |  | J01XX - Other antibacterials | 0 | 0.0 | 0 | 0.0 |

# Supplementary Figures

Supplementary Figure 1. Percentage of total animals affected for each ATCvet group, by third level, for each continent in reports of potential antibiotic resistance in EudraVigilance Veterinary, up to February 21^st^, 2024.
